# Graphene nanosheet-grafted double-walled carbon nanotube hybrid nanostructures by two-step chemical vapor deposition and their application for ethanol detection

**DOI:** 10.1038/s41598-019-44315-y

**Published:** 2019-05-27

**Authors:** Worawut Muangrat, Winadda Wongwiriyapan, Shingo Morimoto, Yoshio Hashimoto

**Affiliations:** 1 Institute of Carbon Science and Technology, Shinshu University, 4-17-1 Wakasato, Nagano, 380-8553 Japan; 2 College of Nanotechnology, King Mongkut’s Institute of Technology Ladkrabang, Chalongkrung road, Ladkrabang, Bangkok 10520 Thailand

**Keywords:** Nanoscience and technology, Sensors

## Abstract

Here, we present a facile technique for synthesis of graphene nanosheet (GNS)-grafted double-walled carbon nanotube (DWCNT) hybrid carbon nanostructures (here after referred to as G-DWCNTs) by directly growing GNSs along the sidewalls of DWCNTs using a two-step chemical vapor deposition (CVD). DWCNTs were synthesized by floating catalyst CVD at 1300 °C using ferrocene and thiophene dissolved in ethanol. Then, GNSs were grafted onto the synthesized DWCNT bundles by thermal CVD at 1300 °C using ethanol. The sharp-edged petal-like structure of GNSs were grown along the sidewalls of DWCNT bundles while maintaining the one-dimensional structure of DWCNT. Next, DWCNTs and G-DWCNTs were dispersed in ethanol, then deposited on the paper using vacuum filtration method and used for ethanol detection. G-DWCNTs sensor exhibited a 3-fold improvement in the response to ethanol vapor compared to the DWCNTs sensor. The sensing mechanism of DWCNTs and G-DWCNTs can be described in terms of charge transfer between the gas molecules and sensing material. These results demonstrate that the facile technique by two-step CVD method provides a promising approach for simple and low-cost technique to synthesize the hybrid nanostructure of GNSs and DWCNTs. The new hybrid carbon nanostructures are attractive for gas sensing application.

## Introduction

Graphene^1,2^ and carbon nanotubes (CNTs)^3,4^ are attractive materials in low-dimensional carbon allotropes which have been received widespread attention due to their unique structure and fascinating properties, such as mechanical^5–7^, electrical^8,9^, and thermal properties^5,6,10^. Graphene and CNTs become an attractive material for research area in the field of nanotechnology. In recent years, the combining between CNTs and graphene have attracted increasing attention because hybrid graphene-CNTs structure possesses the outstanding properties of both two-dimensional graphene and one-dimensional CNTs. Graphenated carbon nanotubes (G-CNTs) are one of the promising of graphene-CNTs three-dimensional hybrid nanostructure that consists of petal-like graphene grown on the sidewalls of CNTs. The advantage of G-CNT hybrid nanostructures is the high surface area of three-dimensional structure. In addition, the G-CNTs have the high edge density of graphene which provide higher charge density and reactivity than the basal plane. Due to their unique structure and outstanding properties, G-CNTs have been widely applied in the areas of the supercapacitor^11–13^, gas sensor^14^, electronic and optoelectronic devices^14^, and proton exchange membrane fuel cells^15,16^. To synthesize G-CNTs, microwave plasma-enhanced chemical vapor deposition (MPECVD) has been considered as a promising method. There have been several reports on the synthesis of G-CNTs using one- or two-step MPECVD^11–20^. For instance, Yu *et al*.^14^ synthesized the G-CNTs by two-steps MPECVD. Multi-walled CNTs (MWCNTs) were grown by the decomposition of acetylene. Then, a few-layer graphene (FLG) were directly grown on the sidewall of MWCNTs using methane. The gas sensor fabricated from MWCNTs-FLG enabled 3-fold improved nitrogen dioxide detection compared with pristine MWCNTs. In addition, the MWCNTs-FLG structure exhibits remarkable optoelectronic property. The one-step MPECVD technique for synthesis of G-CNTs using methane as carbon source was proposed by Henry *et al*.^19^. The G-CNTs exhibited the catalytic activity and electron transfer kinetics two orders magnitude higher and faster than that of CNTs. Recently, there is a report of synthesis of G-CNTs by two-step CVD procedure consisting of thermal CVD (TCVD) and radio frequency plasma-enhanced CVD (RF PECVD)^15,16^. In the first step, MWCNTs were grown directly onto the catalyzed carbon paper by the TCVD using ethylene. In the second step, the G-CNT hybrid was fabricated by growth of graphene directly onto the CNTs without any additional catalyst by the RF PECVD technique using acetylene. The G-CNT-supported platinum (Pt) catalyst exhibits a superior electrochemical stability over that of the carbon black-supported Pt catalyst due to the high crystallinity of the G-CNT support. Although the MPECVD has been considered as a promising method for the synthesis of G-CNT hybrid nanostructures, this technique requires expensive vacuum apparatuses and plasma systems. Moreover, the surface of CNT may deteriorate during the high-energy plasma process, thus the PECVD method can produce the hybridization of GNSs with only MWCNTs. It is still challenging to achieve a hybridization of GNSs and thin-walled CNTs, i.e., double-walled CNTs (DWCNTs).

In this work, we have proposed the facile synthesis of hybrid nanostructures of graphene nanosheet (GNS) grafted double-walled CNT (DWCNT) bundles (hereafter referred to as G-DWCNTs) by two-step CVD method. First, DWCNTs were synthesized by floating catalyst CVD (FCCVD) method, and subsequently GNSs were deposited on the DWCNTs bundles by TCVD method. The structural evolution between GNS and DWCNT bundles are also demonstrated at different deposition times in the TCVD method. The proposed G-DWCNT hybrid nanostructures show figures of merit over the previous reports in terms of the new hybrid carbon nanostructures and simplicity in synthesis method. In addition, synthesized DWCNTs and G-DWCNTs were used as gas sensor for ethanol detection at parts per million (ppm) levels under room temperature. The fabricated sensor from G-DWCNTs enabled an approximately 3-fold improvement in ethanol detection compared to DWCNTs. The improvement in the sensor response of G-DWCNTs is attributed to the GNS-grafted on DWCNT bundles, which provides higher surface area for gas adsorption.

## Results

### Characterization of hybrid carbon nanostructures: morphology, structure and crystallinity

**Figure 1:**
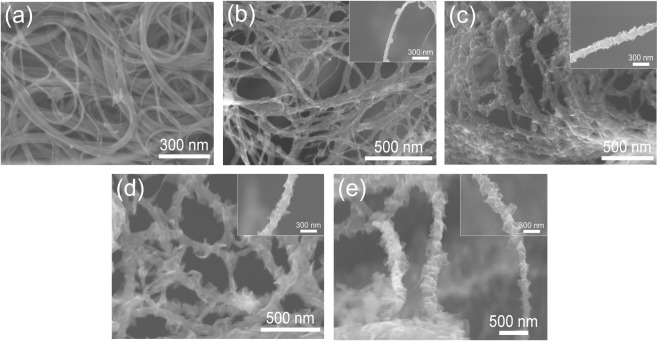
FESEM images of (**a**) DWCNTs, (**b**) G(2.5)-DWCNTs, (**c**) G(5)-DWCNTs, (**d**) G(10)-DWCNTs, (**e**) G(20)-DWCNTs. The inset is a high magnification images.

**Figure 2:**
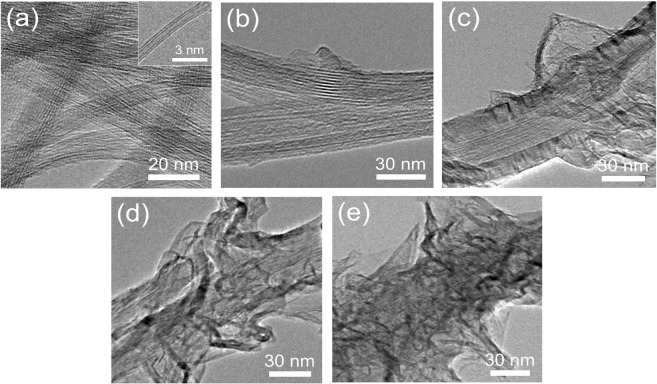
TEM images of (**a**) DWCNTs, (**b**) G(2.5)-DWCNTs, (**c**) G(5)-DWCNTs, (**d**) G(10)-DWCNTs, and (**e**) G(20)-DWCNTs.

Figure 1 shows the FESEM images of DWCNTs and G-DWCNTs. The DWCNTs assembled into the bundles. The diameter of DWCNT bundles are in the range from 10 to 60 nm as shown in Fig. 1(a). For hybrid carbon nanostructures, the structural evolution of G-DWCNT hybrid nanostructure as a function of deposition time in the TCVD method at 2.5, 5, 10 and 20 min was demonstrated in Fig. 1(b–e) (hereafter referred to as G(2.5)-DWCNTs, G(5)-DWCNTs, G(10)-DWCNTs and G(20)-DWCNTs, respectively). The inset in Fig. 1(b–e) displays the isolated G-DWCNT hybrid nanostructures at different deposition times of GNSs. After GNS growth for 5 min, the GNSs start merging on the surface of networked DWCNT bundles, which can obviously observe at the isolated G-DWCNTs (inset of Fig. 1(b)). The petal-like graphene obviously grafted along on the networked DWCNT bundles with increasing the deposition time of GNSs in the TCVD method (inset of Fig. 1(c–e)). For G(5)-DWCNTs and G(10)-DWCNTs, GNSs almost completely deposited along the surface of DWCNT bundles (inset of Fig. 1(c,d)). In the case of G(20)-DWCNTs, DWCNT bundles were completely covered by the layers of GNSs (inset of Fig. 1(d)). These results show that the amount and density of GNSs on the DWCNT bundles strongly depend on the growth time of GNSs in the TCVD method. Figure 2(a–e) shows TEM images of the synthesized DWCNT bundles, G(2.5)-DWCNTs, G(5)-DWCNTs, G(10)-DWCNTs, and G(20)-DWCNTs, respectively. The TEM images reveal DWCNTs in a bundle structure with a well alignment of an individual tube to each other (Fig. 2(a)). The inset of Fig. 2(a) shows TEM image of the individual DWCNT with two graphene layers stacked in parallel on each side. The average diameters of inner and outer tubes of DWCNTs were 0.97 ± 0.24 and 1.78 ± 0.31 nm, respectively. The interlayer spacing of DWCNTs are in the range of 0.34–0.41 nm. After GNS deposition, the petal-like structure of graphene was grafted along the sidewalls of DWCNT bundles as shown in Fig. 2(b–e). At the beginning stage, the DWCNT bundles were deposited by carbon layer, which derived from the decomposition of ethanol (Fig. 2(b)). The decomposed carbon atom deposited on the surface of DWCNT bundles, subsequently graphene sheet started growing on the carbon layer-deposited DWCNT bundles (Fig. 2(b)). It seems that the carbon layer plays an important role as an active layer for GNSs growth on the DWCNT bundles. These results correspond with a previous work which GNSs can grow on the amorphous carbon layer^21,22^. The thickness of amorphous carbon layer was estimated to be 20–30 nm. The existence of amorphous carbon as an interface layer enables growth of the GNSs without catalyst. These interesting phenomena will be discussed later in terms of growth mechanism. Figure 2(b–e) shows that the amount and density of GNSs obviously increased with increasing the deposition time in the TCVD method. The longer deposition time, the more precipitated carbon atoms on the DWCNTs to form GNSs, resulting in a higher amount and density of GNSs on the DWCNT surface.

**Figure 3:**
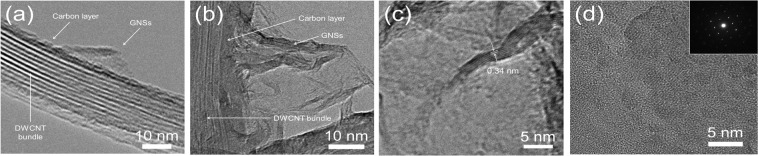
TEM images of interface between GNSs and DWCNT bundles of (**a**) G(2.5)-DWCNTs and (**b**) G(20)-DWCNTs. (**c**) TEM image showing that the interlayer spacing of GNSs of G(20)-DWCNTs. (d) TEM iamge of GNSs; inset shows SAED diffraction pattern of GNSs.

**Figure 4:**
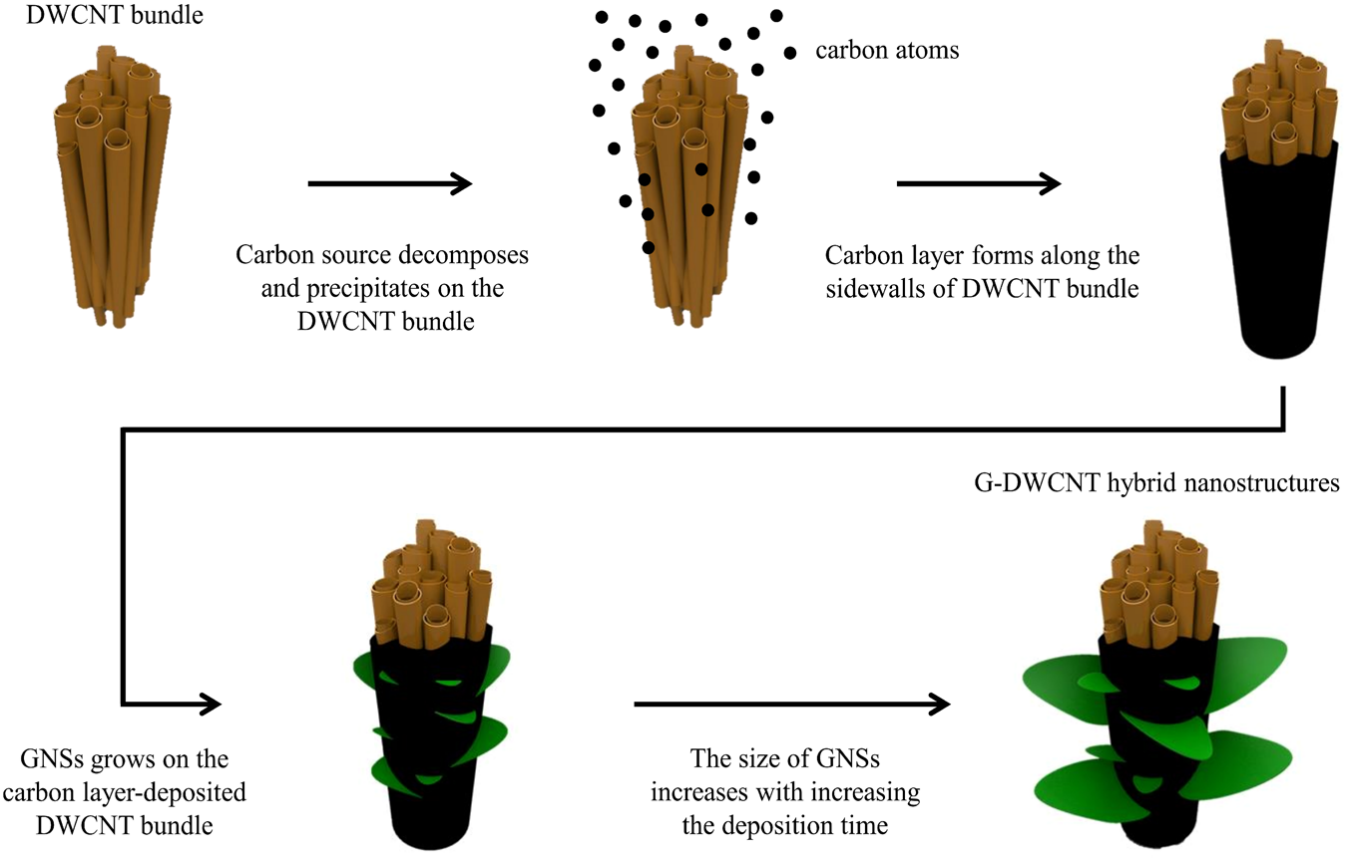
Schematic view of the structural evolution of G-DWCNT hybrid nanostructures.

Figure 3(a,b) shows TEM images of interface between GNSs and DWCNT bundles of G(2.5)-DWCNTs and G(20)-DWCNTs, respectively. Interestingly, it seems that the DWCNT bundles were firstly deposited by carbon layer, followed by the growing of GNSs on the carbon layer-deposited DWCNT bundles (Fig. 3a). We believe that carbon layer acts as intermediated layer between GNSs and DWCNT bundles as clearly seen in Fig. 3(b). In this case, the thickness of intermediated carbon layer is approximately 20 nm. The average size of graphene sheet of G(20)-DWCNTs is in the range of 45 to 130 nm. Figure 3(c) shows a magnified TEM image of G(20)-DWCNTs with several graphene layers oriented parallel to each other, which consists of 4–12 parallel graphene sheets with the range of thickness of 1.1–4.2 nm. The interlayer spacing of GNSs is in the range of 0.34–0.36 nm, which corresponds to the 002 lattice planes of the crystal graphite^23^. These results implying the high crystallinity of hybrid carbon nanostructures between GNSs and DWCNT bundles. Figure 3(d) shows a high-magnification TEM image of a high-ordered regularity orientation of graphene sheet. Selected area electron diffraction (SAED) pattern (inset of Fig. 3(d)) clearly observed the set of six-fold symmetric diffraction pattern with sharp spots. These results confirm the existence of a typical hexagonal crystalline structure of graphene^24^. Figure 4 shows a schematic diagram of the structural evolution of G-DWCNT hybrid nanostructures. We envisioned that the growth mechanism of G-DWCNT hybrid nanostructures can be described as follows: (i) after carbon source exposures into the reaction zone in the TCVD process, carbon source decomposes and precipitates on the surface of DWCNT bundles, (ii) resulting in a formation of carbon layer deposited on the surface of DWCNT bundles: (iii) GNSs start growth on the carbon layer-deposited DWCNT bundles, leading to a formation of the G-DWCNT hybrid nanostructures: (iv) The size of GNSs increases with increasing the deposition time. We suggest that the size of GNSs deposited on the DWCNT bundles can be controlled by the deposition time in the TCVD condition.

**Figure 5:**
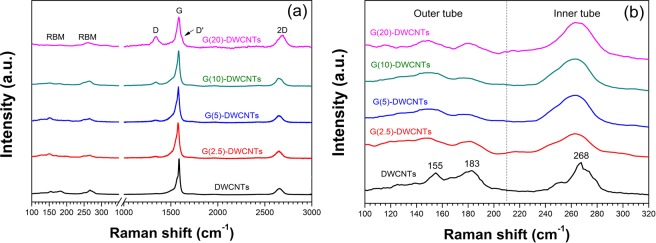
(**a**) Raman spectra and (**b**) RBM spectrum of DWCNTs and G-DWCNTs.

Raman spectroscopy was employed to characterize the structure, purity and crystallinity of DWCNTs and G-DWCNTs. Figure 5(a) shows four significant Raman peaks generated from the G-DWCNTs: DWCNTs-derived radial breathing mode (RBM) at ∼150–27 cm^−1^, disorder carbon-derived D-band at ∼134 cm^−1^, graphitic structure-derived G-band at ∼159 cm^−1^ and second-order of D-band-derived 2D-band at ∼267 cm^−1^. In addition, the spectrum of G(20)-DWCNTs shows the D’ band at 1620 cm^−1^, which originate from the intravalley double resonance process only in presence of defects^25–27^. The position of D-band, G-band and 2D-band and the intensity ratio between the D- and G-bands (*I*_D_/*I*_G_) and the 2D- and G-bands (*I*_2D_/*I*_G_) of DWCNTs and G-DWCNTs are summarized in the Table 1. The *I*_D_/*I*_G_ is an indication of the crystallinity^20,28^ of DWCNTs and G-DWCNTs. The calculated *I*_D_/*I*_G_ ratio of DWCNTs was 0.03. After GNSs deposition, the *I*_D_/*I*_G_ ratios of G(2.5)-DWCNTs, G(5)-DWCNTs, G(10)-DWCNTs and G(20)-DWCNTs were 0.05, 0.07, 0.11, and 0.37, respectively. The *I*_D_/*I*_G_ ratio of G-DWCNTs increased with increasing the deposition time of GNSs, implying that the purity and crystallinity of G-DWCNTs were deteriorated due to the reconstructed between GNSs and DWCNTs, indicating the hybridization of GNSs and DWCNTs, specially for G(20)-DWCNTs which is consistent with the TEM results with the highest amount of GNSs (Fig. 2(e)). The *I*_D_/*I*_G_ value indicates that the G-DWCNTs remain a high-quality graphitic crystalline structure. The *I*_2D_/*I*_G_ ratios of DWCNTs, G(2.5)-DWCNTs, G(5)-DWCNTs, G(10)-DWCNTs and G(20)-DWCNTs were 0.16, 0.18, 0.19, 0.22 and 0.44, respectively. The increase in the *I*_2D_/*I*_G_ ratio suggests the presence of more graphene growth on the DWCNT surface^18,29^. Figure 5(b) shows RBM spectra of DWCNTs and G-DWCNTs. The DWCNTs display two distinctly RBM peaks indicating the presence of inner and outer tubes of DWCNTs. The diameter of the DWCNTs can be determined using ω_RBM_ = 234/d_t_ + 10, where ω_RBM_ and d_t_ are the Raman shift of RBM peak (cm^−1^) and tube diameter (nm), respectively^29,30^. The calculated diameters of DWCNTs from RBM spectra at 155, 183, and 268 cm^−1^ were 1.61, 1.35, and 0.91 nm, respectively. The calculated inner and outer diameters of DWCNTs are in the same range as that observed from the TEM results. After GNSs deposition on DWCNT bundles, the RBM intensity of outer diameter significantly decreased with increasing the TCVD reaction time. The decrease in the RBM intensity of outer diameter could be attributed to the higher amount of GNSs deposition on the DWCNT bundles, resulting in a decrease in a vibration mode in the RBM intensity of outer tube.

**Table 1:** The positions of D-band, G-band and 2D-band and *I*_D_/*I*_G_ and *I*_2D_/*I*_G_ ratios of DWCNTs and G-DWCNTs.

Sample	Peak position (cm^−1^)			Ratio	
	D-band	G-band	2D-band	*I*_D_/*I*_G_	*I*_2D_/*I*_G_
DWCNTs	1340	1587	2655	0.03	0.16
G(2.5)-DWCNTs	1337	1577	2651	0.05	0.18
G(5)-DWCNTs	1343	1581	2652	0.07	0.19
G(10)-DWCNTs	1347	1583	2653	0.11	0.22
G(20)-DWCNTs	1345	1587	2696	0.37	0.41

### Gas sensing characteristics

**Figure 6:**
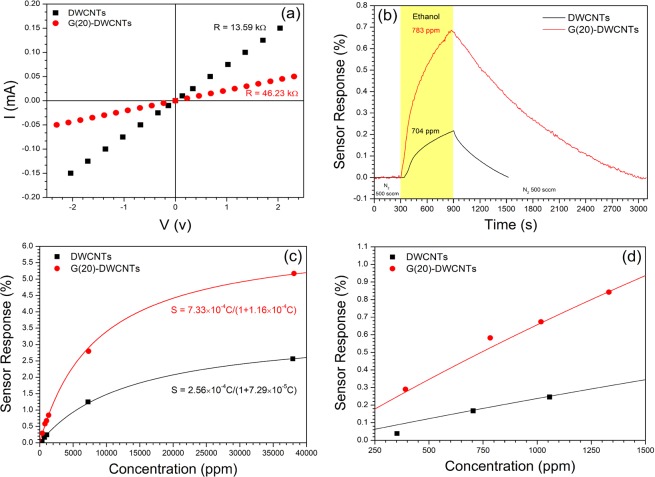
(**a**) *I-V* characteristics of DWCNTs and G(20)-DWCNTs. (**b**) Sensor response as a function of time for DWCNTs and G(20)-DWCNTs toward ethanol vapor. Sensor response of DWCNTs and G(20)-DWCNTs as a function of ethanol concentration at (**c**) high and (**d**) low concentrations.

Figure 6(a) shows *I-V* curves of the DWCNT and G(20)-DWCNT sensors with silver contact electrodes. The *I-V* curves exhibited linear behavior, implying that the contact between DWCNT and G(20)-DWCNT papers and electrodes is Ohmic contact. The initial resistances of DWCNT and G-DWCNT sensors are 13.59 and 46.23 kΩ, respectively. The initial resistance of G-DWCNTs shows a slight increase from that of DWCNTs, which corresponds to the *I*_D_/*I*_G_ value (Table 1). Although the initial resistance of G-DWCNTs increase, the resistance and *I*_D_/*I*_G_ value of G-DWCNTs are similar to those of the DWCNTs. These results implying that the G-DWCNTs remain a high-quality structure. Figure 6(b) shows the sensor responses of DWCNT and G-DWCNT sensors toward ethanol as a function of time under alternating supply of ethanol and N_2_ gas. The electrical resistances of DWCNT and G-DWCNT sensors increased after ethanol exposure and completely recovered to the initial electrical resistance after purging with N_2_ gas. Ethanol molecules are likely to physically adsorb onto the sensing material via van der Waals forces, which is a weak interaction. Thus, ethanol molecules were easily removed by N_2_ gas purging. The G(20)-DWCNT sensor exhibited a 3.2-fold improvement in the response to ethanol vapor compared to the DWCNT sensor. The enhancement of sensor response could be derived from the high surface area of GNS-grafted DWCNTs which provide the area for molecule adsorption. These results are similar to previous work which MWCNTs-FLG sensor enabled 3-fold improvement in nitrogen dioxide detection compared to pristine MWCNTs sensor. Regarding the response time of the sensor, the sensor response time is defined as the time taken to reach 90% of its maximum sensor response. The sensor response of DWCNTs and G-DWCNTs were 480 and 428 s. These results show that the G-DWCNT sensor shows a merit for enhancement and improvement of sensor response magnitude and response time, respectively. The sensing mechanism of DWCNTs and G-DWCNTs can be explained by charge transfer between the adsorbed gas molecules and sensing materials. Upon ethanol adsorption, electron charge transfer from ethanol molecules to DWCNTs and GNSs, decreasing the density of holes in the p-type DWCNTs and p-type GNSs^14,31–34^, resulting in an increase in the electrical resistance.

To investigate the quantitative correlation between sensor response and ethanol concentration, the sensor response of DWCNTs and G(20)-DWCNTs were measured as a function of ethanol concentration (solid squares and circles) in the range of 352–38, 115 ppm as shown in Fig. 6(c). The responses of DWCNTs and G(20)-DWCNTs increase with increasing the ethanol concentration, and tend to saturate at a concentration higher than 35,000 ppm, because the sensing material surface is completely covered by ethanol molecule. The quantitative measurement of ethanol concentration from the sensor response is an important issue for its practical application. The feature of ethanol adsorption in this sensing system can be described based on the Langmuir isotherm as shown in Eq. (1) ^35–37^:

1 $$\theta =\frac{KC}{1+KC}$$

where *θ* is the monolayer coverage of gas molecules on sensor surface, *K* (*ppm*^−1^) is the equilibrium constant for gas adsorption and *C* (*ppm*) is gas adsorption concentration. *θ* is assumed to be proportional to the sensor response, that is, *θ* = *S*/*S*_*max*_ where, *S*_*max*_ is the maximum response of the sensor at saturation coverage. Thus, *C* is given by Eq. (2):

2 $$S=\frac{CK{S}_{max}}{1+KC}$$

The *K* values of DWCNTs and G(20)-DWCNTs were estimated to be 7.29 ×10^−5^ and 1.16 × 10^−4^ ppm^−1^, while *S*_*max*_ values of DWCNTs and G(20)-DWCNTs were 3.51 and 6.32, respectively. The results show that the experiment data has a good fitting to Langmuir isotherm fitting curve (solid lines in Fig. 6(c)), implying that the sensor response of DWCNTs and G(20)-DWCNTs are based on Langmuir adsorption isotherm. Next, we focused on gas detection at a low concentration as shown in Fig. 6(d). The relationships between the sensor response and ethanol concentration are linear, which corresponds to the Langmuir adsorption isotherm at the low concentration range.

Next, the limit of detection (*LOD*) of DWCNTs and G(20)-DWCNTs were investigated. 30 data points prior to exposure to ethanol were plotted and fitted by a fifth-order polynomial. The ${V}_{{x}^{2}}$ was calculated by Eq. (3):

3 $${V}_{{x}^{2}}={\sum }^{}{({y}_{i}-y)}^{2}$$

where *y*_*i*_ is the measured value of the sensor response baseline and *y* is the corresponding value calculated from the fifth-order polynomial fit. Then, the *rms*_*noise*_ of the sensor was calculated by Eq. (4):

4 $$rm{s}_{noise}=\sqrt{\frac{{V}_{{x}^{2}}}{N}}$$

where *N* is the number of data points used for the curve fitting (*N* = 30). Finally, the LOD can be derived from Eq. (5) by the slope of the linear fit on the sensor response versus concentration plot^38^.

5 $$LOD=3\frac{rm{s}_{noise}}{slope}$$

The calculated *LOD* of the G(20)-DWCNTs and DWCNTs and are approximately 13 and 257 ppm, respectively. The LOD of the G(20)-DWCNTs shows an approximately 20-fold higher than that of DWCNTs. We envision that the sensor based on G-DWCNTs could be down part per billion level for ethanol detection by functionalization of G-DWCNTs with metal nanoparticle^31,39,40^.

The facile technique by two-step CVD proposed in this research affords an advantage to grow the hybrid graphene and thin CNT such as DWCNTs while maintains its pristine nanostructure. Moreover, compare to the previous reported methods, such as the plasma-enhanced CVD^11–20^, our technique shows a benefit in terms of easy set-up without a requirement of vacuum system. Furthermore, from the growth mechanism aspects, this facile two-step CVD technique enables the hybrid structures of GNSs with other types of carbon nanostructure, organic and inorganic nanomaterials, providing novel properties emergent at the nanoscale. Furthermore, the synthesized new hybrid G-DWCNTs can be further used as gas sensor for VOC detection.

## Conclusions

The hybrid carbon nanostructures consist of GNSs and DWCNT bundles were successfully synthesized by a simple CVD method. DWCNTs were synthesized by FFCVD method at 1300 °C using ferrocene, thiophene and ethanol. Then, GNSs were grown on the carbon layer-deposited DWCNT bundles by TCVD method at 1300 °C method using ethanol. The deposition time in the TCVD method plays an important role on the amount and density of foliated graphene on the sidewalls of DWCNT bundles. The growth mechanism of G-DWCNT hybrid nanostructures could be described by the carbon layer-deposited on the DWCNT bundle, which acts as the intermediate between GNSs and DWCNT bundles. These results demonstrate the promising hybrid carbon nanostructures grown by facile CVD with simplicity and low-cost techniques. In addition, G(20)-DWCNTs exhibited 3-fold improvement in the sensor response to ethanol compared to DWCNTs. The features of sensor response of DWCNTs and G(20)-DWCNTs can be described by the Langmuir adsorption isotherm. The sensing mechanism of DWCNTs and G-DWCNTs can be described in terms charge transfer between the gas molecules and sensing material. These results demonstrate that G-DWCNT hybrid nanostructures can use as gas sensing device for ethanol detection. Furthermore, these structures can be further used in a wide range of application in the areas of electronic and energy-related devices, and so on.

## Methods

### Synthesis of hybrid carbon nanostructures

DWCNTs were synthesized by FCCVD method using ferrocene (98%, Aldrich) and thiophene (99%, ACROS ORGANICS) dissolved in ethanol solution (99.5%, Nacalai Tesque) as metal catalyst and carbon source, respectively. All substances were mixed by sonication for 30 min before using in the synthesis process. A stainless steel mesh was used as substrate, which was placed at the outlet of alumina tube. At the beginning of the reaction, argon (Ar) gas at a flow rate of 500 standard cubic centimeters per minute (sccm) was introduced into CVD system until the temperature was raised up to 1300 °C. Then, the homogenous mixed solution was injected into reaction zone of CVD system by micropump at a pumping speed of 0.04 mL/min. At the same time, hydrogen (H_2_) gas at a flow rate of 1000 sccm served as carrier gas was introduced into CVD system. The reaction was carried out for 20 min at atmospheric pressure. The as-synthesized samples were continuously grown and formed in the reaction zone, and immediately transferred to deposit on the stainless steel mesh at the outlet. Finally, the as-synthesized samples were cooled down to room temperature under Ar atmosphere at a flow rate of 500 sccm. The as-synthesized samples were further purified by acid and air-annealing treatments repeatedly for 2 times. The as-synthesized samples were soaked in 37% hydrochloric (HCl) acid for 24 h, then filtered and washed with distilled water until pH 7. Next, the samples were annealed under air atmosphere at 400 °C for 30 min in order to remove the amorphous carbon and the metal catalysts. Then, the samples were again washed with HCl and, subsequently annealed under air atmosphere at 500 °C for 30 min. Next, the purified DWCNTs were placed at the middle reaction zone of CVD to graft GNSs on DWCNTs using ethanol as carbon source. The temperature was raised up to 1300 °C under Ar gas at a flow rate of 500 sccm. The ethanol solution was injected into reaction zone of CVD system by micropump at a pumping speed of 0.04 mL/min using H_2_ gas at a flow rate of 1000 sccm. The CVD synthesis was carried out for 2.5–20 min at atmospheric pressure. Finally, the hybrid carbon nanostructures were cooled down to room temperature under Ar atmosphere at a flow rate of 500 sccm.

### Characterization techniques

The morphologies and nanostructure of the DWCNTs and G-DWCNT hybrid nanostructures were characterized by field emission scanning electron microscopy (FESEM, Hitachi SU8000) and transmission electron microscopy (TEM, JEOL JEM-2100) operated at 10 and 200 kV, respectively. Raman spectroscopy (Horiba Jobin Yvon) was employed to characterize the crystallinity and purity using a wavelength of 532 nm (2.33 eV). The current-voltage (*I-V*) characteristics were measured in the voltage range of −2 to 2 V by a source measurement unit.

### Fabrication of sensor devices and gas sensor measurement

To investigate potential use as sensor, ethanol detection using DWCNTs and G-DWCNTs was demonstrated. DWCNTs and G(20)-DWCNTs were separately sonicated with ethanol at the concentration of 0.01 mg/mL for a designed time period. The dispersions of DWCNTs and G(20)-DWCNTs were separately vacuum filtered by using a polytetrafluoroethylene (PTFE) membrane (Advantec, pore size of 1.0 µm) and dried at 100 °C for 60 min. The DWCNTs and G(20)-DWCNTs on PTFE were cut in to a size of 1 × 2 cm^2^. Then, the copper wires were contacted on the sensor by silver paste. The distance between two electrodes was 1 cm, thus the sensing area was 1 × 1 cm^2^. The sensor response to ethanol vapor was investigated at room temperature by recording their electrical resistance using digital multimeter (Keithley, 2450). The fabricated sensors from DWCNTs and G(20)-DWCNTs were placed in a stainless steel chamber and then N_2_ gas was introduced into the chamber at a flow rate of 500 sccm for 5 min as a baseline. Then, ethanol vapor prepared from bubbling liquid ethanol with 5–500 sccm of nitrogen (N_2_) gas to measure the sensor response of DWCNT and G(20)-DWCNT sensors. The detection was carried out for 10 min. The ethanol concentration is in the range of 352–38, 115 ppm. For recovery, the sensor was recovered by N_2_ gas at a flow rate of 500 sccm until its initial electrical resistance. The sensor response (*S* (%)) of all sensors was calculated using the Eq. (6):

6 $$S\,( \% )=\frac{{R}_{Ethanol}-{R}_{{N}_{2}}}{{R}_{{N}_{2}}}\times 100$$

where *R*_*Ethanol*_ and ${R}_{{N}_{2}}$, are the electrical resistances of the sensor after and before ethanol exposure, respectively. The schematic view of the sensor measurement system is shown in the supporting information.

## Supplementary information

Figure S1
