# Supplementary material for: Graphene nanosheet-grafted double-walled carbon nanotube hybrid nanostructures by two-step chemical vapor deposition and their application for ethanol detection
Source: Sci Rep. 2019 May 27;9:7871. doi: 10.1038/s41598-019-44315-y (PMC6536556; doi:10.1038/s41598-019-44315-y)
Supplement: Supplementary file 1 — Figure S1 [file 41598_2019_44315_MOESM1_ESM.docx]

Supplementary information for

Graphene nanosheet-grafted double-walled carbon nanotube hybrid nanostructures by two-step chemical vapor deposition and their application for ethanol detection

Worawut Muangrat,^a^ Winadda Wongwiriyapan, ^b^ Shingo Morimoto ^a^ and

Yoshio Hashimoto ^a^

^a^ Institute of Carbon Science and Technology, Shinshu University, 4-17-1 Wakasato, Nagano, 380-8553, Japan

^b^ College of Nanotechnology, King Mongkut’s Institute of Technology Ladkrabang, Chalongkrung road, Ladkrabang, Bangkok 10520, Thailand


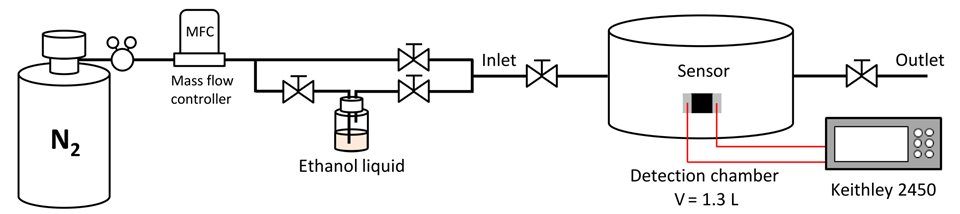


**Fig. S1.** The schematic view of the sensor measurement system.
